# Supplementary material for: Allograft Inflammatory Factor-1 in Metazoans: Focus on Invertebrates
Source: Biology (Basel). 2020 Oct 24;9(11):355. doi: 10.3390/biology9110355 (PMC7692721; doi:10.3390/biology9110355)
Supplement: Supplementary file 1 [file biology-09-00355-s001.pdf]

biology

Allograft Inflammatory Factor-1 in Metazoans: Focus on Invertebrates

Jacopo Vizioli [1], Tiziano Verri [2] and Patrizia Pagliara [2,*]

[1] Inserm, Univ.Lille, Inserm, U1192—Protéomique Réponse Inflammatoire Spectrométrie de Masse—PRISM, F-59000 Lille, France; jacopo.vizioli@univ-lille.fr

[2] Dipartimento di Scienze e Tecnologie Biologiche e Ambientali, Università del Salento, Via Provinciale Lecce-Monteroni, 73100 Lecce, Italy; tiziano.verri@unisalento.it

* Correspondence: patrizia.pagliara@unisalento.it

Received: 31 August 2020; Accepted: 21 October 2020; Published: date

Percent Identity Matrix - created by Clustal2.1

1: A_japonica|XP_033099142.1|crinoids	100.00	56.29	58.28	54.30	56.95	42.57	44.37	43.05	43.45	44.06	40.82	39.46	43.62	51.66	49.01	50.00	51.05	55.70	50.99	51.06	53.85	53.10	47.95	48.63	50.00	50.00	51.03	52.45	50.00	50.00
2: P_lividus|MN128715.1|sea_urchins	56.29	100.00	60.93	52.98	59.60	43.24	45.03	41.06	40.69	45.45	41.50	40.82	48.99	51.66	49.01	49.32	52.45	54.36	53.64	53.90	51.05	50.34	47.95	48.63	47.95	45.21	46.90	52.45	50.68	51.39
3: O_echinata|GAUQ01047814.1|brittle_stars	58.28	60.93	100.00	60.93	62.91	45.27	53.64	46.36	41.38	49.65	46.94	44.22	50.34	59.60	56.95	55.41	53.15	59.73	52.32	55.32	51.05	60.69	56.85	59.59	58.22	56.16	57.93	58.04	54.79	56.25
4: A_japonicus|HADF01043973.1|sea_cucumbers	54.30	52.98	60.93	100.00	64.24	44.59	50.33	45.03	40.69	41.26	42.18	40.82	46.98	52.98	50.99	49.02	47.30	55.70	51.66	51.77	48.95	56.55	55.48	57.53	55.48	55.48	57.24	57.34	54.11	56.25
5: A_planci|XP_022085355.1|starfish	56.95	59.60	62.91	64.24	100.00	49.32	50.99	45.03	44.83	50.35	46.26	43.54	53.69	56.29	54.97	50.68	53.85	59.73	56.95	59.57	56.64	60.69	58.22	58.90	60.27	57.53	59.31	58.04	57.53	61.81
6: C_gigas|XP_019918396.1|bivalves	42.57	43.24	45.27	44.59	49.32	100.00	57.43	55.41	44.83	48.25	48.98	46.26	56.76	54.73	49.32	45.95	54.55	47.30	50.68	52.48	49.65	53.79	52.74	54.11	53.42	54.11	55.17	54.55	56.16	53.47
7: M_galloprovincialis|AJQ21490.1|bivalves	44.37	45.03	53.64	50.33	50.99	57.43	100.00	62.91	51.03	52.45	51.02	51.02	57.05	57.62	57.62	56.08	62.94	52.35	56.29	59.57	55.24	59.31	59.59	60.27	59.59	60.96	59.31	56.64	54.79	55.56
8: A_farreri|ABY84846.1|bivalves	43.05	41.06	46.36	45.03	45.03	55.41	62.91	100.00	48.28	45.45	46.26	45.58	49.66	50.99	49.01	52.03	54.55	46.98	49.01	51.06	51.75	51.72	54.11	56.85	54.79	54.11	54.48	53.15	49.32	47.92
9: E_lucius|NP_001297949.1|bony_fishes	43.45	40.69	41.38	40.69	44.83	44.83	51.03	48.28	100.00	63.89	64.83	64.83	45.52	51.03	46.90	51.37	47.92	48.97	52.41	52.82	48.25	55.17	50.34	51.72	53.10	51.03	51.72	55.24	52.41	50.69
10: G_japonicus|XP_015277931.1|lizards	44.06	45.45	49.65	41.26	50.35	48.25	52.45	45.45	63.89	100.00	74.13	69.93	51.05	55.94	54.55	51.39	53.47	52.45	56.64	59.15	53.85	58.04	55.94	57.34	56.64	55.24	56.64	59.44	56.64	58.74
11: H_sapiens|NP_001614.3|mammals	40.82	41.50	46.94	42.18	46.26	48.98	51.02	46.26	64.83	74.13	100.00	89.12	49.66	53.74	52.38	51.02	51.75	48.98	54.42	55.32	50.35	56.55	54.79	56.85	54.11	56.85	55.86	57.34	54.79	52.78
12: M_musculus|NP_062340.1|mammals	39.46	40.82	44.22	40.82	43.54	46.26	51.02	45.58	64.83	69.93	89.12	100.00	47.62	51.02	48.98	48.98	50.35	46.26	52.38	53.90	48.95	53.79	52.05	53.42	50.68	52.74	51.03	54.55	52.05	50.00
13: A_californica|XP_005111045.2|gastropods	43.62	48.99	50.34	46.98	53.69	56.76	57.05	49.66	45.52	51.05	49.66	47.62	100.00	66.44	67.11	47.97	56.64	53.02	54.36	52.48	48.95	55.86	56.16	57.53	58.22	58.22	58.62	55.24	56.16	56.25
14: H_diversicolor|JU064069.1|gastropods	51.66	51.66	59.60	52.98	56.29	54.73	57.62	50.99	51.03	55.94	53.74	51.02	66.44	100.00	81.46	52.70	57.34	55.70	62.25	58.87	52.45	61.38	60.96	63.70	65.07	61.64	62.76	62.24	58.22	61.11
15: H_discus_discus|ACJ65689.1|gastropods	49.01	49.01	56.95	50.99	54.97	49.32	57.62	49.01	46.90	54.55	52.38	48.98	67.11	81.46	100.00	51.35	55.94	53.02	62.91	56.74	49.65	60.00	58.90	60.96	61.64	60.27	61.38	58.74	56.16	59.72
16: H_medicinalis|AHZ89388.1|segmented_worms	50.00	49.32	55.41	49.02	50.68	45.95	56.08	52.03	51.37	51.39	51.02	48.98	47.97	52.70	51.35	100.00	72.00	52.70	60.81	56.34	54.55	58.62	59.59	64.38	58.22	56.85	59.31	64.34	60.96	61.11
17: L_rubellus|GIFV01025258.1|segmented_worms	51.05	52.45	53.15	47.30	53.85	54.55	62.94	54.55	47.92	53.47	51.75	50.35	56.64	57.34	55.94	72.00	100.00	53.85	65.03	59.86	58.04	61.54	60.84	62.24	61.54	61.54	63.64	64.34	58.74	61.54
18: S_kowalevski|FF529285.1|hemichordates	55.70	54.36	59.73	55.70	59.73	47.30	52.35	46.98	48.97	52.45	48.98	46.26	53.02	55.70	53.02	52.70	53.85	100.00	54.36	60.28	52.45	60.00	57.53	59.59	58.22	54.11	57.24	60.84	60.27	59.03
19: P_dumerilii|GBZT01000129.1|segmented_worms	50.99	53.64	52.32	51.66	56.95	50.68	56.29	49.01	52.41	56.64	54.42	52.38	54.36	62.25	62.91	60.81	65.03	54.36	100.00	59.57	55.94	60.69	60.96	63.01	60.96	61.64	62.07	63.64	61.64	61.81
20: S_rosetta|XP_004998111.1|choanoflagellates	51.06	53.90	55.32	51.77	59.57	52.48	59.57	51.06	52.82	59.15	55.32	53.90	52.48	58.87	56.74	56.34	59.86	60.28	59.57	100.00	65.96	66.67	61.70	61.70	66.67	61.70	62.41	63.83	66.67	65.25
21: M_brevicollis_MX1|XP_001746044.1|choanoflagellates	53.85	51.05	51.05	48.95	56.64	49.65	55.24	51.75	48.25	53.85	50.35	48.95	48.95	52.45	49.65	54.55	58.04	52.45	55.94	65.96	100.00	67.13	60.84	62.24	62.24	62.24	64.34	61.54	61.54	64.34
22: E_pallida|XP_020916259.1|sea_anemones	53.10	50.34	60.69	56.55	60.69	53.79	59.31	51.72	55.17	58.04	56.55	53.79	55.86	61.38	60.00	58.62	61.54	60.00	60.69	66.67	67.13	100.00	82.43	83.78	85.81	81.76	83.78	74.13	75.17	77.08
23: A_viridis|Cuttitta_et_al_2017|sea_anemones	47.95	47.95	56.85	55.48	58.22	52.74	59.59	54.11	50.34	55.94	54.79	52.05	56.16	60.96	58.90	59.59	60.84	57.53	60.96	61.70	60.84	82.43	100.00	94.63	86.58	84.56	83.11	73.43	71.92	72.92
24: A_tenebrosa|XP_031560363.1|sea_anemones	48.63	48.63	59.59	57.53	58.90	54.11	60.27	56.85	51.72	57.34	56.85	53.42	57.53	63.70	60.96	64.38	62.24	59.59	63.01	61.70	62.24	83.78	94.63	100.00	86.58	84.56	84.46	75.52	74.66	74.31
25: A_digitifera|XP_015755194.1|stony_corals	50.00	47.95	58.22	55.48	60.27	53.42	59.59	54.79	53.10	56.64	54.11	50.68	58.22	65.07	61.64	58.22	61.54	58.22	60.96	66.67	62.24	85.81	86.58	86.58	100.00	87.25	85.81	74.13	73.29	79.17
26: S_pistillata|XP_022794071.1|stony_corals	50.00	45.21	56.16	55.48	57.53	54.11	60.96	54.11	51.03	55.24	56.85	52.74	58.22	61.64	60.27	56.85	61.54	54.11	61.64	61.70	62.24	81.76	84.56	84.56	87.25	100.00	91.22	74.83	71.23	77.08
27: O_faveolata|XP_020615825.1|stony_corals	51.03	46.90	57.93	57.24	59.31	55.17	59.31	54.48	51.72	56.64	55.86	51.03	58.62	62.76	61.38	59.31	63.64	57.24	62.07	62.41	64.34	83.78	83.11	84.46	85.81	91.22	100.00	74.83	73.10	79.17
28: H_caerulea|GFSI01017565.1|sponges	52.45	52.45	58.04	57.34	58.04	54.55	56.64	53.15	55.24	59.44	57.34	54.55	55.24	62.24	58.74	64.34	64.34	60.84	63.64	63.83	61.54	74.13	73.43	75.52	74.13	74.83	74.83	100.00	76.22	81.82
29: C_stipitata|GHWA01007108.1|sponges	50.00	50.68	54.79	54.11	57.53	56.16	54.79	49.32	52.41	56.64	54.79	52.05	56.16	58.22	56.16	60.96	58.74	60.27	61.64	66.67	61.54	75.17	71.92	74.66	73.29	71.23	73.10	76.22	100.00	82.64
30: S_domuncula|CAC38780.1|sponges	50.00	51.39	56.25	56.25	61.81	53.47	55.56	47.92	50.69	58.74	52.78	50.00	56.25	61.11	59.72	61.11	61.54	59.03	61.81	65.25	64.34	77.08	72.92	74.31	79.17	77.08	79.17	81.82	82.64	100.00

Figure S1. Percent Identity Matrix (upper panel) created by Clustal Omega alignment (lower panel). Amino acid sequence alignment of metazoan AIF-1. Multiple sequence alignment was generated using Clustal Omega at https://www.ebi.ac.uk/Tools/msa/clustalo/ using default parameters.
